# Supplementary material for: Learning curve and surgical outcome of robotic assisted colorectal surgery with ERAS program
Source: Sci Rep. 2022 Nov 29;12:20566. doi: 10.1038/s41598-022-24665-w (PMC9709162; doi:10.1038/s41598-022-24665-w)
Supplement: Supplementary file 1 — Supplementary Information 1. [file 41598_2022_24665_MOESM1_ESM.zip › LSC Raw data-Ñ[▒K/080/N_ñw╝╨░O▒Kñσ.pdf]

請選擇...reporter

臺 中 榮 民 總 醫 院

手術室護理記錄

[redacted]

基本資料

- 室別： 二醫手術室-31室，手術科別： CRS，手術類別： 常規，日期： 2020/06/03
- 進入時間： 1105 ； 麻醉開始時間： 1130 ； Time Out時間： 1210 ； 劃刀時間： 1211 ； 手術結束時間： 1650 送出時間： 1655 ；
- 麻醉方式： GE

  診斷： Malignant tumor of sigmoid colon 40 cm. GBS

  手術名稱： 1.Laparoscopic Anterior resection and anastomosis - malignant
  2.Laparoscopic Cholecystectomy

  手術局部用藥： nil

  術前預防性抗生素：
  - 抗生素名稱一： Veterin 1GM IVD
  - 時間： 1155
  - 上次簽章: 戴宛榛

  ，追加預防性抗生素：
- 手術醫師： 林俊余，吳峰旭，林昌霖，洪宇漢
- 麻醉醫師： VS 施涵薰
-
  - 刷手護士A ： 吳雅華，時間： 1105-1230，接替刷手一： 王毓均，時間： 1230-1655
  - 流動護士A ： 戴宛榛，時間： 1105-1300，接替流動一： 吳雅華，時間： 1300-1500，接替流動二： 戴宛榛，時間： 1500-1655

皮膚狀況

A-V Shunt： 有

術前： 不完整

- 2.瘀傷
- 7. 造口

部位(術前)：

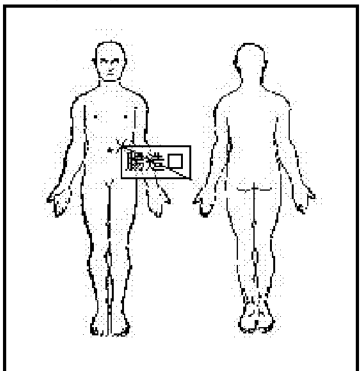

左側腸造口;雙手多處瘀青

記錄者： 上次簽章: 戴宛榛

術後： 不完整

部位(術後)：

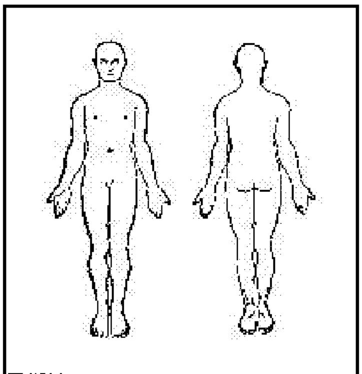
同術前

記錄者： 上次簽章: 戴宛榛

手術準備

手術臥式 單一手術臥式

-
    - 手術臥式： 截石臥式
    - 使用附件： 矽膠枕頭，腳架
    - 皮膚準備： 10%Aq Betadine，Hibiscrub，其它： 2% Chlorhexidine
    - 電燒： 使用電燒: 電極片位置： 其它： 迴路電極墊，記錄者： 上次簽章: 戴宛榛

止血帶T： 無

植入物

- 植入物名稱/號碼/數量/部位： RELOAD ENDOCUTTER 60MM-4.1MM/*2/大腸
  Hyalobarrier gel endo/10ml/*1/腹部

, 記錄者： 上次簽章: 戴宛榛

標本送檢

- 細菌培養 一般，採取部位: 1. Gallbladder，數量： 壹
- 細胞學 無
- 冰凍切片 無
- 病理標本
  組織名稱一： IMA Root
  ,
  組織名稱二： Distal cutend
  ,
  組織名稱三： Gallbladder
  ，更多組織名稱
    - 組織名稱四： S-colon
- 截肢 無

標本交付 標本： 肆 袋，標本： 零 盒，標本： 零 瓶，細胞學： 零 管，Culture Tube： 壹 管，被交付醫師： R 洪宇渼，

- 記錄者： 上次簽章: 戴宛榛
- 時間： 1630

傷口術後敷料

傷口敷料： 傷口敷料： 無

- 請於圖上標示部位：

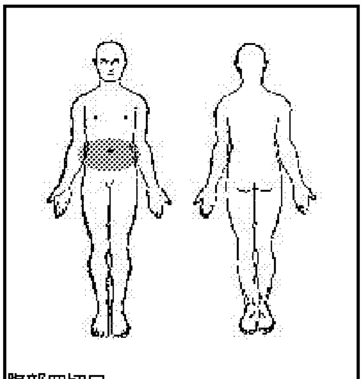

- 傷口敷料： Meplor

導管

導管	天數	導管資訊	插入日期	插入時間	更換日期	拔除日期	拔除時間	拔除原因	預計拔除/更換	預計拔除/更換日期
Sump NG Tube(1)	1	號碼:16 Fr.; 深度:52CM; 部位:胃;	2020/06/03	1130						
Foley(1)	1	號碼:16 Fr.; 部位:膀胱;	2020/06/03	1130						
Penrose Drain(1)	1	號碼:12 mm.; 部位:肝下; 註記:上;	2020/06/03	1600						
Penrose Drain(1)	1	號碼:12 mm.; 部位:骨盆腔; 註記:下;	2020/06/03	1600						
A-line (1)	1	號碼:20 G; 部位:前臂; 象限:LU;	2020/06/03	1150						

用物計數

- 手術中出血量： Minimal c.c.
- 手術中尿量： 100 c.c.
-
    - 其它： 無

用物計數： 單一切口

- 1.器械： 正確，2.縫針： 正確，3.刀片： 正確，4.紗布： 正確，5.腹部墊： 正確，6.紗球： 未使用，7.PEANUTS ： 未使用，8.紗條： 未使用，9.絲帶： 未使用，10.針頭： 未使用，11.Vessel loop： 未使用，12.Boots： 未使用，13.Jelco： 未使用，14.棉條： 未使用，
    - 第一次：
        - 刷手護士 ： 王毓均
        - 流動護士： 上次簽章: 吳雅華
        - 時間： 1450

  ,
    - 第二次：
        - 刷手護士 ： 王毓均
        - 流動護士： 上次簽章: 戴宛榛
        - 時間： 1610

  ,
    - 第三次：
        - 刷手護士 ： 王毓均
        - 流動護士： 上次簽章: 戴宛榛
        - 時間： 1620

備註

備註：NIL

- 記錄者： 上次簽章: 戴宛榛
- 時間： 1615

填寫護理人員 戴宛榛

正式摘要

請選擇...reporter

doctorRank:B
